# Exploring the Significance of the Exon 4-Skipping Isoform of the ZNF217 Oncogene in Breast Cancer

**DOI:** 10.3389/fonc.2021.647269

**Published:** 2021-07-02

**Authors:** Aurélie Bellanger, Diep T. Le, Julie Vendrell, Anne Wierinckx, Lőrinc S. Pongor, Jérôme Solassol, Joël Lachuer, Philippe Clezardin, Balázs Győrffy, Pascale A. Cohen

**Affiliations:** ^1^ Université Lyon 1, Lyon, France; ^2^ CRCL-Centre de Recherche en Cancérologie de Lyon-Inserm U1052-CNRS U5286, Lyon, France; ^3^ INSERM, UMR1033 LYOS, Lyon, France; ^4^ Département de Pathologie et Oncobiologie, Laboratoire de Biologie des Tumeurs Solides, CHU Montpellier, Univ. Montpellier, Montpellier, France; ^5^ ProfileXpert, SFR-Est, CNRS UMR-S3453, INSERM US7, Lyon, France; ^6^ Department of Bioinformatics, Semmelweis University, Budapest, Hungary; ^7^ TTK “TermészetTudományi Kutatóközpont” Momentum Cancer Biomarker Research Group, Institute of Enzymology, Budapest, Hungary; ^8^ Institut de Recherche en Cancérologie de Montpellier (IRCM), INSERM U1194, Univ. Montpellier, Montpellier, France

**Keywords:** ZNF217, isoform, splice variant, prognosis, breast cancer

## Abstract

Oncogene alternative splicing events can create distinct functional transcripts that offer new candidate prognostic biomarkers for breast cancer. ZNF217 is a well-established oncogene but its exon 4-skipping isoform (ZNF217-ΔE4) has never been investigated in terms of clinical or biological relevance. Using *in silico* RNA-seq and RT-qPCR analyses, we demonstrated for the first time the existence of *ZNF217-ΔE4* transcripts in primary breast tumors, and a positive correlation between *ZNF217-ΔE4* mRNA levels and those of the wild-type oncogene (*ZNF217-WT*). A pilot retrospective analysis revealed that, in the Luminal subclass, the combination of the two *ZNF217* variants (the *ZNF217-ΔE4-WT* gene-expression signature) provided more information than the mRNA expression levels of each isoform alone. Ectopic overexpression of ZNF217-ΔE4 in breast cancer cells promoted an aggressive phenotype and an increase in ZNF217-WT expression levels that was inversely correlated with DNA methylation of the *ZNF217* gene. This study provides new insights into the possible role of the ZNF217-ΔE4 splice variant in breast cancer and suggests a close interplay between the ZNF217-WT and ZNF217-ΔE4 isoforms. Our data suggest that a dual signature combining the expression levels of these two isoforms may serve as a novel prognostic biomarker allowing better stratification of breast cancers with good prognosis and aiding clinicians in therapeutic decisions.

## Introduction

Breast cancer is the most frequent cancer in women, with an estimated 2.2 million new cases being diagnosed in 2020 worldwide, and the leading cause of cancer death in women (> 684,000 cases in 2020) (1). The *ZNF217* gene is located on chromosome 20q13.2, a region frequently amplified in many tumors, including those of the breast (2). The encoded protein is an oncogenic transcription factor belonging to the Krüppel-like zinc finger transcription factors family. Previous studies highlighted that the ZNF217 oncogene is involved in both early and late stages of tumor progression [for review (3)]. Our previous work contributed to the demonstration that breast cancer cells possessing high ZNF217 expression levels display a more aggressive phenotype (*e.g.* increased cell proliferation, increased invasive properties and resistance to chemotherapy) (4, 5). High *ZNF217* mRNA levels in primary breast tumors are also of bad prognosis and associated with shorter relapse free survival (RFS) and metastasis development (5, 6), with the most discriminatory prognostic power observed in Luminal breast cancers (7).

Alternative splicing is now considered as a hallmark of cancer (8), participating in tumor progression through the expression of distinct isoforms. Key splicing events targeting important factors in tumorigenesis have been identified in breast cancer (*e.g., TP53, HER2, BRCA1, FGFR, BIRC5/survivin,.*). Alternative splicing events (ASE) in breast cancer thus represent candidate biomarkers and candidate therapeutic targets [for review (9)]. Considering the ZNF217 oncogene, only little is known about the existence of its splice variants. In the original study discovering the human *ZNF217* gene (2), the *in silico* alignment of cDNAs from colon carcinoma and HeLa cell lines suggested a possible alternative processing of the 133-base pairs exon 4. The corresponding putative exon 4-skipping *ZNF217* (*ZNF217-ΔE4*) mRNA would encode for a ZNF217 protein isoform with a C-terminus sequence [1013-1061 amino acids (aa)] distinct from that of ZNF217 wild-type (ZNF217-WT) protein (1013-1048 aa sequence) (2). Nevertheless, since the original description (2), the *ZNF217-ΔE4* variant has never been studied, either in terms of expression in human tumor samples or in terms of biological relevance.

Considering the key role of the ZNF217 oncogene in breast cancer and its strong biomarker value, the *ZNF217-ΔE4* transcript is thus of utmost interest. The main objectives of the present work were to newly investigate: (i) whether *ZNF217-ΔE4* is expressed in primary breast cancers; (ii) if so, whether *ZNF217-ΔE4* mRNA levels possess any clinical relevance; and (iii) whether increased expression levels of ZNF217-ΔE4 functionally affect the phenotype of breast cancer cells.

By performing *in silico* RNAseq and real-time quantitative polymerase chain reaction (RT-qPCR) investigations, our study highlights the existence of *ZNF217-ΔE4* transcripts in primary breast tumors. Interestingly, *ZNF217-ΔE4* mRNA levels were significantly correlated with those of *ZNF217-WT* in tumor samples. A pilot retrospective analysis revealed that, in the Luminal subclass, the combination of the two ZNF217 variants (the ZNF217-ΔE4-WT gene-expression signature) provided more information than the mRNA expression levels of each isoform alone. Strikingly, the ectopic overexpression of ZNF217-ΔE4 in breast cancer cells led to: (i) a more aggressive phenotype, similar to that previously observed with ZNF217-WT (4, 5); (ii) increased expression of endogenous ZNF217-WT; and (iii) decreased DNA methylation on key CpG sites within the *ZNF217* gene.

## Materials and Methods

### Analysis of RNA-seq Data

A total of 1,097 RNA-seq aligned BAM files were obtained from the TCGA repository (10). Samples were filtered to include only primary tumor samples in women. The ratio of the exon 4 skipping isoform of the ZNF217 gene in each sample was calculated based on the number of reads that had spliced alignments from exon 3 to exon 4 junction (E3-E4) compared to all reads mapped to the exon 3 to exon 5 junction (E3-E5). The ratio was used to quantify the isoform expression from the DESeq2 normalized data for each sample separately. Expression for exon 3 was computed using the following formula: Exon_3_DESeq = (Exon_3_reads/Total_reads)* Gene_DESeq. A minimal expression of 1,000 was used as a cutoff to designate patients as those positive for the ZNF217 exon 4-skipping isoform. Kruskal-Wallis test was used to compare breast cancer subclasses distribution. The correlation of expression was tested using the Pearson correlation coefficient. Statistical significance was set at *p* < 0.05.

### *ZNF217* Isoforms Plasmid Constructs

The pcDNA6-ZNF217-WT plasmid has been previously described and established (4). The pcDNA6 plasmid (Invitrogen, Cergy Pontoise, Paris) containing ZNF217 exon4 skipping isoform DNA sequence (pcDNA6-ZNF217-ΔE4) was produced as follows: the DNA sequence specific for *ZNF217-ΔE4* was obtained after polymerase chain reaction (PCR) amplification from breast cancer cells using specific primers, then ligated into the restriction enzymes pre-digested pcDNA6-ZNF217-WT plasmid to replace the DNA region specific for *ZNF217-WT* with that of *ZNF217-ΔE4*.

### RT-qPCR Amplification of *ZNF217* Isoforms

Total RNA extraction, reverse transcription, and RT-qPCR measurements were performed as described previously (4, 5). CFX96 equipment with the SsoAdvanced Universal SYBR green supermix (BioRad, Hercules, USA) was designed for RT-qPCR measurements, according to the manufacturer’s recommendations. Specific primers were used to amplify the DNA sequence for *ZNF217* isoforms. The 5′-AGTCCAAATCCCTGCCATCT-3′ and 5′-GGGGAAACACTGGTTTTAGG-3′ primers amplify a region within exon 3 (E3). The 5′-CTCGACGTTAGAAGGAAAAAG-3′ and 5′-TGGTCGATAATGTGCATTCC-3′ primers were used to explore *ZNF217*-WT (exon 3 – exon 4 junction, E3-E4). The 5′-GTGGCTGACTGTTCAGAAGCCC-3′ and 5′-GACATCCACCAAGACCTTCTA-3′ primers are specific for the ZNF217-ΔE4 isoform (exon 3 – exon 5 junction, E3-E5). For investigation in breast tumor samples and MDA-MB-231 stable transfectants, all measurements were normalized to the ribosomal *28S* gene expression using the 5′-CGATCCATCATCCGCAATG-3′ and 5′-AGCCAAGCTCAGCGCAAC-3′ primers.

### Primary Breast Tumor Cohort

Women with primary breast tumors (n = 107) and known clinical follow-up who had not received any therapy before surgery and who relapsed, or not, while receiving endocrine therapy and/or chemotherapy were recruited from the BB-0033-00050, Biological Resources Center (CRB) Centre Léon Bérard, Lyon France (**Supplementary Table 1**). This study has been approved by the local ethics committee (CRB Centre Léon Bérard, France). The CRB Centre Léon Bérard is quality certified according to NFS96-900 French standard and ISO 9001 for clinical trials, ensuring scientific rigor for sample conservation, traceability, and quality, as well as ethical rules observance and defined rules for transferring samples for research purposes (Ministry of Health for activities authorization n◦ AC-2019-3426 and DC-2008-99). The material used in the study has been collected in agreement with all applicable laws, rules, and requests of French and European government authorities, including the patients’ informed written consents. Extraction of total RNA from frozen tumor samples and RT-qPCR measurements were performed as previously described (5). Univariate (log-rank) analysis, multivariate analysis and all statistical analyses were performed using the SPSS™ Software (IBM, USA). For univariate analyses, the data were divided at the median value of *ZNF217-E3, ZNF217-WT (E3-E4)*, or *ZNF217-ΔE4 (E3-E5)* mRNA expression into two groups with either high or low expression levels. A *p* < 0.05 was considered statistically significant.

### Establishment of MDA-MB-231- ZNF217-ΔE4 Stable Transfectants

MDA-MB-231 breast cancer cells were grown according to recommendations in DMEM medium supplemented with 10% fetal bovine serum (Invitrogen). MDA-MB-231 cells were stably transfected with pcDNA6 or pcDNA6-ZNF217-ΔE4 plasmids, then selected in the presence of 20 µg/ml blasticidin. MDA-MB-231-ZNF217-WT cells have been previously established (4, 5).

### Western Blotting

Western blot experiments were performed as previously described (5) with a commercial anti-ZNF217 antibody produced against a peptide comprised within the 1000-1048 aa sequence of ZNF217-WT (#48133; Abcam, Paris, France), a home-made rabbit polyclonal antibody directed against the C-terminus 1045-1060 aa sequence specific for ZNF217-ΔE4 isoform (RM217 Ab, Covalab, Lyon, France), the home-made polyclonal Cov-2 antibody directed against the C-terminus 1035-1048 aa sequence specific for ZNF217-WT isoform (Covalab) and anti-α-tubulin antibody (#T5168; Sigma, St Louis, MO, USA).

### Cell Proliferation

Cells (20,000 cells per well) were plated in triplicates into a 24-well plate then grown up to 96h. The medium was changed every 2 days. Proliferating cells were analyzed using the Scepter™ 2.0 Cell Counter (Merck Millipore, Billerica, USA).

### Cytotoxicity Assay

As previously described (4), cells (8,000 cells per well) were plated onto a 96-well plate, treated for 4 days with 10^-12^ to 10^-6^ M of paclitaxel (Paxene^®^, Ivax, Miami, USA). Cell viability was assessed with the CellTiter 96 AQueous One Solution Cell Proliferation assay (Promega, Madison, WI, USA).

### Soft-Agar Colony-Formation Assay

The experiment was performed as previously described using 10,000 cells (11). Fifteen days later, the cells were stained with 0.005% Cristal violet (Sigma-Aldrich) for 1 h.

### Methylome

For each cell line, genomic DNA was extracted from two independent cell cultures using the QIAamp DNA mini kit (Qiagen, Hilden, Germany). The Illumina Infinium MethylationEPIC Beadchip was used to obtain DNA methylation profiles according to the manufacturer’s instructions. Briefly, 500 ng of gDNA (QuantiFluor^®^ dsDNA System, Promega) were bisulfite-modified using EZ-DNA Methylation-Gold Kit (Zymo Research) and then used for the genome-wide methylation assays. The iDat’s files were obtained by scanning the array on iScan scanner (Illumina).

### Pyrosequencing Analysis

Genomic DNA (500 ng) was used in bisulfite conversion reactions with the EpiTect^®^ Bisulfite Kit (Qiagen), according to the manufacturer’s protocol. The bisulfite converted DNA was amplified using the PyroMark PCR kit (Qiagen) using the 5′-GTTATTTGTTAAGAAGTGAAGGAAT-3′ and the 5′-biotin-CCTTACTACTAAATAACTTAAAATC-3′ primers for the two following CpG: chr20:52198378 (cg01692482) and chr20:52198279 (cg00703481). PCR products were used for CpG quantification using a PyroMark Q24 (Qiagen) as the supplier’s recommendation. The percentage of methylation of the two CpG sites was calculated using the PyroMark Q24 Advanced software (Qiagen).

## Results

### *In Silico* RNA-seq Analysis Reveals the Expression of *ZNF217 Exon 4-Skipping* Isoform in Primary Breast Tumor Samples

**Figure 1:**
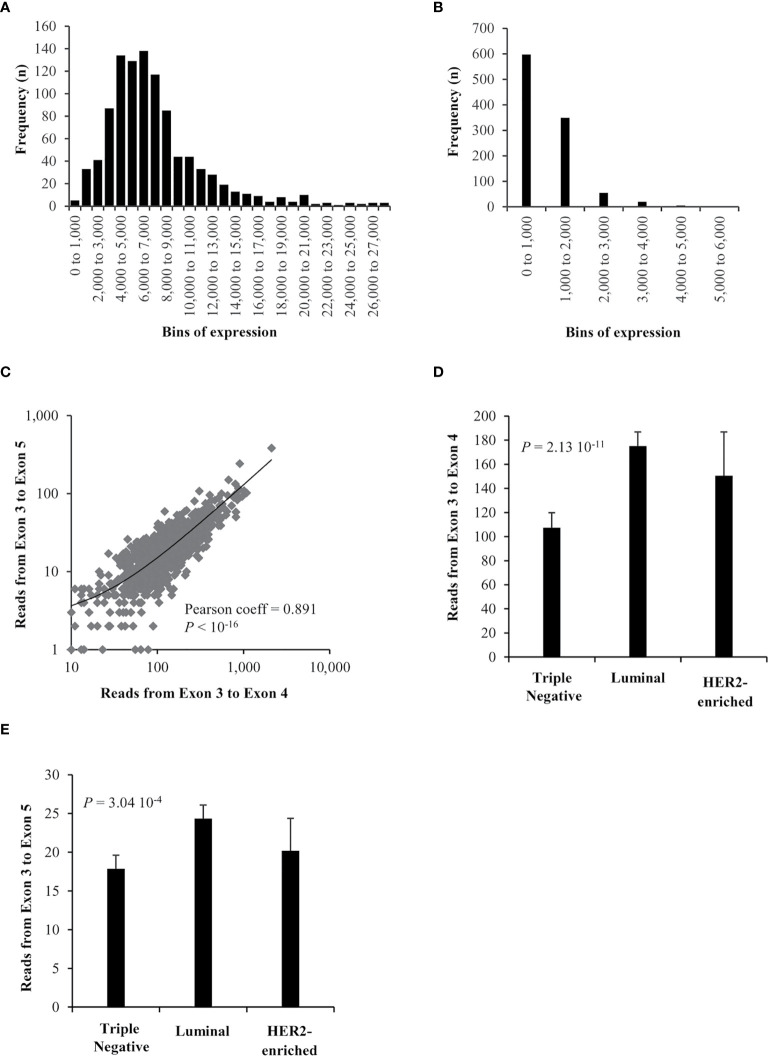
RNAseq expression of *ZNF217* isoforms in breast cancer. **(A)** *ZNF217-WT (E3-E4)* expression and **(B)** *ZNF217-ΔE4 (E3-E5)* expression, in bins of thousands. **(C)** A high correlation was observed when correlating the expression of splice reads mapped to exon 3 – exon 5 junction versus those mapped to exon 3 – exon 4 junction. There is a significant expression difference between breast cancer molecular subtypes of both **(D)** exon 3 –exon 4 reads and **(E)** exon 3 - exon 5 reads.

RNA-seq files obtained from the TCGA repository (10) were used to decipher whether the *ZNF217-ΔE4* isoform is expressed in 1,097 primary breast tumors. Identification of reads mapped to the exon 3 to exon 4 junction (E3-E4) of the *ZNF217* gene corresponds to the expression of *ZNF217-WT* isoform in breast tumor samples. Our analysis further discovered spliced alignments from exon 3 to exon 5 (exon 4-skipping, E3-E5), revealing the expression of the *ZNF217-ΔE4* isoform in primary breast cancers. The distribution of *ZNF217-WT (E3-E4)* and *ZNF217-ΔE4 (E3-E5)* mRNA levels in the primary breast tumors highlighted that the *ZNF217-ΔE4* isoform mRNA expression levels are globally weaker than those of the *ZNF217-WT* isoform (**Figures 1A, B**). *ZNF217-ΔE4* expression levels ranged from 0 to 6,000, with 42.1% of the patients expressing the *ZNF217-ΔE4* isoform over 1,000, while *ZNF217-WT* expression levels ranged from 0 to 27,000 (**Figures 1A, B**). The *ZNF217-ΔE4 (E3-E5)* expression levels represented more than 10% of *ZNF217-WT* expression levels in 76% of the patients (between 10% and 20% in 61.5% of the patients, and higher than 20% in 14.5% of the patients (data not shown)).

Importantly, **Figure 1C** reveals that *ZNF217-WT (E3-E4)* and *ZNF217-ΔE4 (E3-E5)* mRNA levels are significantly positively correlated (Pearson coefficient = 0.891, *p* < 10^-16^). The 1,097 patients were then stratified according to molecular subtypes into Luminal, Triple negative, HER2-enriched subclasses (12–14). **Figures 1D, E** show that both *ZNF217-WT (E3-E4)* and *ZNF217-ΔE4 (E3-E5)* display significant expression differences between the molecular subtypes (*p* = 2.13 10^-11^ and *p* = 3.04 10^-4^, respectively, Kruskal-Wallis test).

Altogether, the RNA-seq data analysis newly demonstrated: (i) the existence of the exon 4-skipping variant of the ZNF217 oncogene in primary breast cancers, expressed at weaker mRNA levels than those of *ZNF217-WT*; (ii) correlated mRNA expression levels of *ZNF217-ΔE4* and *ZNF217-WT* isoforms in primary breast tumor.

### Establishment of Primers for Specific RT-qPCR Investigation of *ZNF217* Isoforms

With the aim of further specifically investigating *ZNF217-ΔE4 (E3-E5)* mRNA expression in clinical samples, three specific primer pairs were designed to discriminate the *ZNF217-WT (E3-E4)* and *ZNF217-ΔE4 (E3-E5)* mRNA expression levels (**Supplementary Figure 1A**). The primer pair 1 amplifies within the sequence of *ZNF217*’s exon 3, a region common to both *ZNF217-WT* and *ZNF217-ΔE4* isoforms. The isoforms thus detected with primer pair 1 will be named *ZNF217-E3*. The primer pair 2 detects the *ZNF217-WT* isoform since the forward primer is located at the E3-E4 junction. The primer pairs 3 detects *ZNF217-ΔE4* isoform, with the reverse primer hybridizing to the E3-E5 junction. The PCR efficiency calculated from standard curves validated that the three primer pairs led to similar and high PCR efficiency values (data not shown).

RT-qPCRs were performed to assess the three primer pairs’ specificity within a complex DNA mixture containing different proportions of pcDNA6-ZNF217-WT and/or pcDNA6-ZNF217-ΔE4 plasmids. **Supplementary Figure 1B** shows a steady number of *ZNF217-E3* copies corresponding to a constant number of pcDNA6-ZNF217 plasmids copies in the mixture. Importantly, primer pair 2 or primer pair 3 were able to discriminate and quantify the number of, respectively, pcDNA6-ZNF217-WT or pcDNA6-ZNF217-ΔE4 copies actually deposited in the mixture (**Supplementary Figure 1B**). Altogether, these results validated that these pairs of primers are specific and sensitive for each isoform, even within a mixture of *ZNF217* isoforms.

### RT-qPCR Validation of *ZNF217-WT* and *ZNF217-ΔE4* Expression in an Independent Cohort of Primary Breast Tumors

We performed RT-qPCR analyses to explore *ZNF217-WT (E3-E4)* and *ZNF217-ΔE4 (E3-E5)* mRNA expression levels in a cohort of 107 human primary breast tumor samples (**Supplementary Table 1**). The patterns of distribution of *ZNF217-WT (E3-E4)* and *ZNF217-ΔE4 (E3-E5)* mRNA levels over the 107 primary breast tumors (**Supplementary Figures 1C, D**) were very similar to those observed in **Figures 1A, B**. Overall, the *ZNF217-ΔE4 (E3-E5)* mRNA levels were 6-8 fold weaker than those of *ZNF217-WT (E3-E4)* within a particular primary breast tumor (data not shown). The *ZNF217-ΔE4 (E3-E5)* mRNA levels represented less than 10% of *ZNF217-WT* expression levels in 25% of the patients, 10-30% in 44% of the patients, and higher than 30% in 31% of the patients (data not shown). In accordance with what we have observed by RNAseq analyses, the RT-qPCR investigation found *ZNF217-WT (E3-E4)* and *ZNF217-ΔE4 (E3-E5)* mRNA levels significantly and positively correlated (Pearson coefficient = 0.807, *p* < 10^-4^). Again, both *ZNF217-WT (E3-E4)* and *ZNF217-ΔE4 (E3-E5)* isoforms display significant mRNA expression differences between the breast cancer molecular subtypes (*p* = 0.007 and *p* = 0.056, respectively, Kruskal-Wallis Test, data not shown).

### The *ZNF217-ΔE4-WT* Gene Expression Signature Indicates Poor Prognosis in Luminal Breast Cancers

We have previously reported that high mRNA levels of *ZNF217* are of poor prognosis and represent an early indicator of relapse in breast cancer, with the most powerful prognostic value observed in Luminal subtypes (5, 7). However, at the time when those studies were conducted, the possible expression of the *ZNF217-ΔE4* isoform in breast tumors was unknown. The primer pair used in our previous studies (5, 7) targets exon 3 of the *ZNF217* gene (primer pair 1, *ZNF217-E3*), thus exploring in reality both ZNF217*-WT* and *ZNF217-ΔE4* mRNA expression levels. Taking advantage of the specificity of primer pair 2 and primer pair 3, we thus aimed at investigating in a pilot study the prognostic value of *ZNF217-WT (E3-E4)* and *ZNF217-ΔE4 (E3-E5)* mRNA expression levels, alone or in combination, in the molecular subtypes of breast cancer.

In accordance with our previous work (5, 7), the present retrospective analysis of the cohort composed of 107 human primary breast tumor samples confirmed that high *ZNF217-E3* mRNA levels: (i) were significantly associated with shorter relapse free survival (RFS) in the whole cohort (*p =* 0.031 **Table 1**) and in the Luminal subclass (*p =* 0.030, **Table 1** and **Supplementary Figure 2A**); (ii) were not informative in the HER2-enriched, or Triple negative subclasses (**Table 1**). In the whole cohort, *ZNF217*-*ΔE4 (E3-E5)* mRNA levels were significantly associated with shorter RFS (*p* = 0.025, **Table 1**). In the Luminal subclass, high mRNA levels of *ZNF217-WT (E3-E4)* and high mRNA levels of *ZNF217*-*ΔE4 (E3-E5)* did not reach significance after 10 years of retrospective analysis, while a tendency could be observed (**Table 1** and **Supplementary Figures 2B, C**). We then constructed a gene expression signature based on *ZNF217-WT (E3-E4)* and *ZNF217-ΔE4 (E3-E5)* mRNA expression levels by dividing patients into three groups: patients who expressed low *ZNF217-*WT *(E3-E4)* and low *ZNF217-ΔE4 (E3-E5)* mRNA levels (group 1); patients who expressed high *ZNF217-WT (E3-E4)* or high *ZNF217-ΔE4 (E3-E5)* mRNA levels (group 2); patients who expressed high *ZNF217-WT (E3-E4)* and high *ZNF217-ΔE4 (E3-E5)* mRNA levels (group 3). **Supplementary Figure 2D** illustrate that: (i) 83.3% (60 out of 72) of the patients in the Luminal subclass belonged either to group 1 or to group 3, again supporting correlated mRNA levels of *ZNF217-*WT and *ZNF217-*ΔE4 in human primary breast tumors; (ii) the resulting Kaplan Meier curves for RFS in Luminal subclasses showed patients’ stratification into diverse prognostic classes (*p* = 0.05), with the patients belonging to group 1 having the better prognosis (and Kaplan Meier curves for RFS for group 2 and group 3 close). Consequently, we further constructed a *ZNF217-WT-ΔE4* gene expression signature based on two new groups: patients who expressed low *ZNF217-WT (E3-E4)* and low *ZNF217-ΔE4 (E3-E5)* mRNA levels; patients who expressed high *ZNF217-WT (E3-E4)* and/or high *ZNF217-ΔE4 (E3-E5)* mRNA levels. Strikingly, the prognostic value of the *ZNF217-WT-ΔE4* gene expression signature for RFS was demonstrated to be significant in the Luminal subclasses (*p =* 0.023, **Table 1** and **Supplementary Figure 2E**), and not informative in the HER2-enriched, or Triple negative subclasses (**Table 1**).

**Table 1:** Univariate analyses (10 years-retrospective analysis) of the *ZNF217* isoforms mRNA expression levels with regards to relapse-free survival.

	All tumor samples			Luminal subclass^d^			HER2-enriched subclass^d^			Triple negative subclass^d^		
	(n = 107)			(ER+ and/or PR+)			(ER-/PR-/HER2+)			(ER-/PR-/HER2-)		
				(n = 72)			(n = 15)			(n = 20)		
	HR^a^	95% CI*^b^*	*p* ^c^	HR^a^	95% CI*^b^*	*p* ^c^	HR^a^	95% CI*^b^*	*p* ^c^	HR^a^	95% CI*^b^*	*p* ^c^
***ZNF217-E3***	4.62	0.85-5.52	0.031	4.72	1.04-13.20	0.030	0.026	0.17-4.57	NS (0.872)	0.024	0.05-12.87	NS (0.876)
**mRNA levels**												
***ZNF217-WT (E3-E4)***	3.32	0.76-4.56	NS (0.068)	2.86	0.82-8.23	NS (0.091)	1.01	0.41-13.63	NS (0.314)	0.001	0.06-15.18	NS (0.970)
**mRNA levels**												
***ZNF217-ΔE4 (E3-E5)***	5.05	0.93-6.05	0.025	3.00	0.84-8.33	NS (0.083)	0.216	0.27-8.21	NS (0.642)	0.161	N/A^e^	NS (0.204)
**mRNA levels**												
***ZNF217-WT-ΔE4***	5.12	0.95-8.29	0.024	5.11	1.07-21.23	0.023	1.54	0.41-30.84	NS (0.214)	1.296	N/A^e^	NS (0.255)
**signature**												

^a^HR, Hazard ratio.

^b^95% CI, 95% confidence interval.

^c^p was considered significant when p < 0.05. NS, not significant.

^d^subclasses of breast cancer were determined (ER, PR, HER2) according to the St Gallen recommendation (12-14).

^e^N/A, not applicable as all the cases are censored in the low ZNF217-ΔE4 (E3-E5) mRNA level and in the low ZNF217-WT-ΔE4 signature groups.

To investigate the clinical relevance of our findings, we also investigated by univariate analysis the classical prognostic parameters usually studied in breast cancer. **Supplementary Table 2** and **Table 1** indicated that in the whole cohort, the prognostic value of *ZNF217-E3* or of *ZNF217-WT-ΔE4* gene expression signature for RFS was more informative than conventional biomarkers (age, SBR grade, Lymph node status, Macroscopic tumor size, ER status, PR status, HER2 status). In the Luminal subclass (ER+ and or PR+), the prognostic value of *ZNF217-E3* mRNA levels or of *ZNF217-WT-ΔE4* gene expression signature for RFS was more informative than age, SBR grade, Lymph node status, Macroscopic tumor size, and HER2 status (**Table 1** and **Supplementary Table 2**). However, ER status and PR status were associated with shorter RFS (*p* = 0.025 and *p* = 0.032, respectively, **Supplementary Table 2**). Prognostic factors for RFS in the Luminal subclass with a 0.05 significance level in univariate analysis were then entered in a multivariate Cox model. When entering *ZNF217-E3* mRNA levels, ER status, PR status in the multivariate analysis, only *ZNF217-E3* mRNA levels persisted in the model (*p* = 0.04, HR = 3.71; 95%CI =1.04-13.18, data not shown). Similarly, when entering *ZNF217-WT-ΔE4* gene expression signature, ER status, PR status in the multivariate analysis, only *ZNF217-WT-ΔE4* gene expression signature persisted in the model (*p* = 0.04, HR = 4.78; 95%CI =1.07-21.23, data not shown). Finally, no association was found between *ZNF217-E3*, *ZNF217-WT, ZNF217-ΔE4* or *ZNF217-WT-ΔE4* gene expression signature with age, invaded lymph nodes, macroscopic tumor size, ER status, PR status and HER2 status (**Supplementary Table 3**). Considering SBR grade, while the p value was significant (*p =* 0.002), the distribution of *ZNF217-WT* mRNA levels did not reflect any association between high *ZNF217-WT* mRNA levels and high grade, or vice versa (**Supplementary Table 3**). With the aim to clarify the association between *ZNF217-WT-ΔE4* gene expression signature and SBR grade (*p =* 0.024, **Supplementary Table 3**), we performed univariate analyses in both SBRI+II (n= 45) and SBRIII (n= 61) subclasses. Strikingly, *ZNF217-WT-ΔE4* gene expression signature was significantly associated with shorter RFS in the SBRI+II subclass (*p* = 0.005, data not shown), but not in the SBRIII subclass (*p* = 0.64, data not shown), indicating that the *ZNF217-WT-ΔE4* gene expression signature could allow the re-stratification of patients with breast cancers of low/intermediate grade.

Altogether, our pilot study suggests that: (i) at least in the Luminal subclass, the *ZNF217-WT-ΔE4* gene expression signature has a better prognostic value than that of *ZNF217-WT* mRNA levels alone, *ZNF217-ΔE4* mRNA levels alone or that of the other conventional prognostic parameters of breast cancer; and (ii) in the Luminal subclass, the prognostic value of the *ZNF217-WT-ΔE4* gene expression signature recapitulates that of *ZNF217-E3* mRNA levels.

### Constitutive Expression of ZNF217-ΔE4 In Breast Cancer Cells Promotes An Aggressive Phenotype

**Figure 2:**
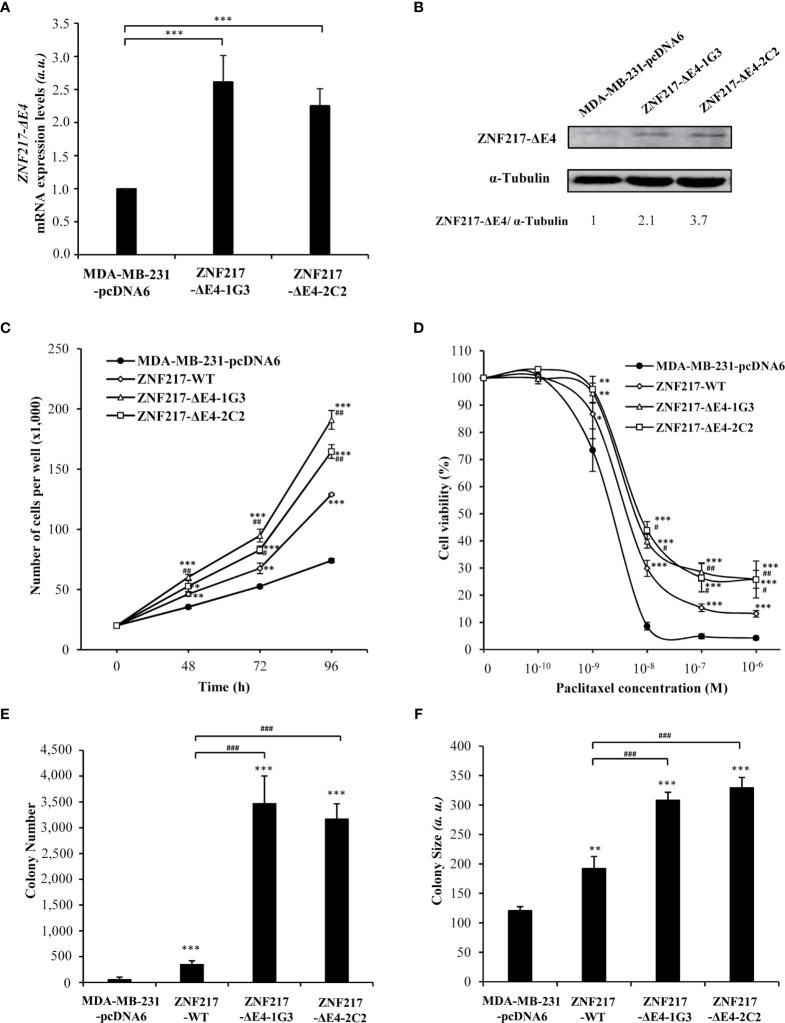
Constitutive expression of *ZNF217-ΔE4* enhances cell proliferation, paclitaxel resistance and anchorage-independent growth of MDA-MB-231 cells. **(A)** RT-qPCR analysis of *ZNF217-ΔE4 (E3-E5)* mRNA expression levels in MDA-MB-231-ΔE4 cell lines and MDA-MB-231-pcDNA6 control cells (mean ± SD from three independent experiments, *a.u.*, arbitrary units). **(B)** Representative Western blot analysis of ZNF217-ΔE4 expression levels in MDA-MB-231-ΔE4 cell lines *versus* control cells using RM217 antibody. **(C)** Cell proliferation assessment of the two MDA-MB-231-ΔE4 cell lines (means ± SD of three independent experiments). **(D)** Cell viability after 96 hours of paclitaxel exposure as assessed by MTS cytotoxicity assay. **(E)** Average number of colonies per well, and **(F)** average size of the colonies in soft agar. (*a.u*.), arbitrary units. Data illustrated in **(E, F)** represent mean ± SD of three independent experiments. In **(A, C–F)**: *p < 0.05, **p < 0.01 and ***p < 0.001 *versus* MDA-MB-231-pcDNA6 cells (Student’s t-test). In **(C–F)**: ^*#*^ *p* < 0.05, ^##^ *p* < 0.01 and ^###^ *p* < 0.001 *versus* ZNF217-WT cells (Student’s t-test).

We have previously described the deleterious effect of *ZNF217-WT* expression after stably transfecting MDA-MB-231 cells, a relevant breast cancer cell model because possessing low endogenous levels of ZNF217 (4, 5). With the aim to study the impact of *ZNF217-ΔE4* expression on breast cancer cells phenotype, we established stable MDA-MB-231 cells constitutively overexpressing the ZNF217-ΔE4 protein. Two cell clones (ZNF217-ΔE4-1G3 and ZNF217-ΔE4-2C2), overexpressing *ZNF217-ΔE4* mRNA (**Figure 2A**) and ZNF217-ΔE4 protein (**Figure 2B**), were selected under blasticidin, and their phenotype was compared with that of MDA-MB-231-pcDNA6 control cells and of previously established ZNF217-WT cells (4, 5). ZNF217-WT cells did not display any significant increase in ZNF217-ΔE4 expression levels (**Supplementary Figure 3**). Our previous work demonstrated that ectopic expression of ZNF217-WT in MDA-MB-231 cells triggers an aggressive phenotype [increased cell proliferation, increased anchorage-independent growth, resistance to paclitaxel (4, 5)], as illustrated in **Figures 2C–F**. We newly found that the constitutive expression of ZNF217-ΔE4 led to a significant increase in proliferation of both ZNF217-ΔE4-1G3 and ZNF217-ΔE4-2C2 cells, compared to MDA-MB-231-pcDNA6 control cells or to ZNF217-WT cells (**Figure 2C**). To investigate whether ZNF217-ΔE4 alters the response to the chemotherapeutic drug paclitaxel, we performed dose-response experiments to measure IC50 values. Strikingly, constitutive expression of ZNF217-ΔE4 led to significant increase in cell viability in the presence of paclitaxel (**Figure 2D**), with a relative resistance of 3.2- and 3.8-fold, respectively, for ZNF217-ΔE4-1G3 and ZNF217-ΔE4-2C2 cells (IC50_MDA-MB-231-pcDNA6_ = 1.8 ± 0.3×10^-9^ M, IC50_ZNF217-ΔE4-1G3_ = 5.7 ± 0.4×10^-9^ M, IC50 _ZNF217-ΔE4-2C2_ = 7.0 ± 1.0×10^-9^ M). The relative resistance developed by the ZNF217-WT cells was of 2.3 (with an IC50 _ZNF217-WT_ = 4.2 ± 0.85×10^-9^ M). Anchorage-independent growth is a phenotype commonly associated with aggressiveness and metastasis. We found that the number and the size of colonies formed in soft agar when ZNF217-ΔE4 was constitutively overexpressed were significantly higher in ZNF217-ΔE4-1G3 and ZNF217-ΔE4-2C2 cells than in control cells or than in ZNF217-WT cells (*p* < 0.001, **Figures 2E, F**). Altogether, these data demonstrate that overexpression of ZNF217-ΔE4 in breast cancer cells enhances their aggressiveness and that the impact of ZNF217-ΔE4 expression on the phenotype of breast cancer cells seems to be at least as or more efficient than that of ZNF217-WT.

### Ectopic Over-Expression of ZNF217-ΔE4 Is Associated With Increased Expression of ZNF217-WT

**Figure 3:**
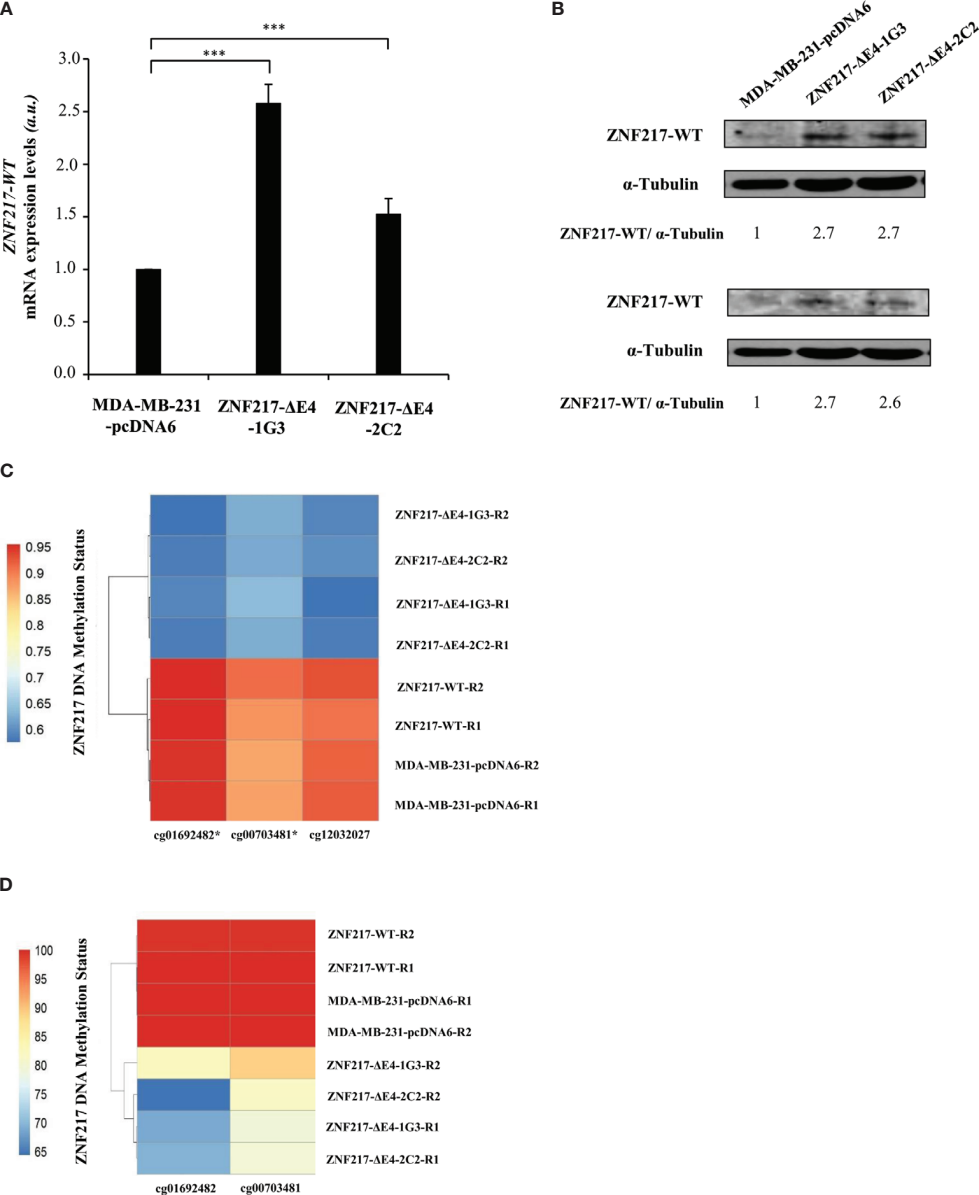
Constitutive *ZNF217-ΔE4* expression leads to increased expression of *ZNF217-WT* and demethylated DNA status of the *ZNF217* gene. **(A)** RT-qPCR analysis of *ZNF217-WT (E3-E4)* mRNA expression levels in MDA-MB-231-ΔE4 cell lines and MDA-MB-231-pcDNA6 control cells (mean ± SD from three independent experiments, *a.u.*, arbitrary units) ***p < 0.001, in Student *t*-test. **(B)** Representative Western blot analysis of ZNF217 WT protein expression levels using Abcam #48133 (upper panel) and Covalab 2 antibodies (lower panel). **(C)** β-values of 3 CpG sites located in the first Exon of the *ZNF217* gene in MDA-MB-231-pcDNA6 control cells, in MDA-MB-231-ZNF217-ΔE4 and in MDA-MB-ZNF217-231-WT cell lines were calculated after genome-wide methylation analysis with Illumina Infinium MethylationEPIC Beadchip. R1 and R2, independent genomic replicates. Heatmaps were realized with pheatmap package (Version: 1.0.12) in RStudio (Version 1.4.1106). Demethylated positions validated by pyrosequencing are indicated by an asterisk**. (D)** Heatmap of pyrosequencing analysis of cg01692482 and cg00703481 in MDA-MB-231-pcDNA6 control cells and in MDA-MB-231-ZNF217-ΔE4 and in MDA-MB-231-ZNF217-WT cell lines. R1 and R2, independent genomic replicates.

Having demonstrated that ectopic and constitutive overexpression of ZNF217-ΔE4 in MDA-MB-231 cells confers a phenotype similar to that previously described when ZNF217-WT is constitutively overexpressed (4, 5), we sought to clarify the expression levels of endogenous ZNF217-WT in ZNF217-ΔE4-1G3 and ZNF217-ΔE4-2C2 cells. Strikingly, both cell lines displayed increased mRNA levels and protein levels of endogenous ZNF217-WT (**Figures 3A, B**). The molecular mechanisms regulating the transcription of the *ZNF217* gene are barely known. We cloned the -2110 base pairs to +300 base pairs region of the *ZNF217* gene into a firefly luciferase reporter plasmid, but we did not observe any increased firefly signal when pcDNA6-ZNF217-ΔE4 plasmid was co-transfected (data not shown), ruling out a positive direct transcriptional regulation by the ZNF217-ΔE4 isoform. Converging epigenetic data stress out that the methylation status at CpG sites within the *ZNF217* locus correlates with inverse *ZNF217* expression levels (7, 15–18). We thus investigated the DNA methylation status at the *ZNF217* CpG sites in both ZNF217-ΔE4-1G3 and ZNF217-ΔE4-2C2 cell lines, in comparison with control MDA-MB-231-pcDNA6 cells Our methylome analyses revealed in both ZNF217-ΔE4-1G3 and ZNF217-ΔE4-2C2 cells (but not in ZNF217-WT cells) the demethylated DNA status of three sites belonging to a CpG island included in exon 1 (GRCh37/hg19) of the *ZNF217* gene: chr20:52198378 (cg01692482), chr20:52198279 (cg00703481), and chr20:52198225 (cg12032027). Heat map visualizing the DNA methylation status at the three identified CpG sites (cg01692482, cg00703481, and cg12032027) illustrates the consensus clustering result, demonstrating DNA demethylated status in both ZNF217-ΔE4-1G3 and ZNF217-ΔE4-2C2 cell lines compared to MDA-MB-231-pcDNA6 control cells or ZNF217-WT cells (**Figure 3C**). Pyrosequencing analyses validated this finding for cg01692482 and cg00703481 (**Figure 3D**). Specific primers targeting cg12032027 could not be designed, thus not allowing to explore this CpG site by pyrosequencing analysis. Altogether, our data highlighted that ectopic overexpression of ZNF217-ΔE4 leads to increased expression on endogenous ZNF217-WT correlated with an inverse DNA methylation status of the *ZNF217* gene.

## Discussion

A growing body of evidence suggests that ASE significantly affect tumor progression, including in the breast. Several studies have indicated that breast cancer-specific ASE include the generation of proteins with new functions (8, 9, 19, 20). Such studies have also underscored the richness of ASE as a source of biomarkers for breast cancer and attempted to define transcript isoform signatures that are associated with molecular subtypes, tumor grade, ER status, or metastasis (8, 9, 19–22).

The first key finding of our study was the identification of *ZNF217-ΔE4* mRNA in primary breast tumors, indicating that the exon 4-skipping process of the *ZNF217* pre-mRNA occurs in breast cancer. This was thoroughly validated by RNAseq or RT-qPCR analyses in primary breast tumors from independent cohorts. Alternative splicing of other members of the Krüppel-like factor (KLF) gene family, including the KLF6 tumor suppressor and the KLF4 regulator of stem cell pluripotency, has been previously described and shown to result in the production of several isoforms with specific biological functions and biomarker value (22–25). Our study thus highlights ZNF217 as a further member of the KLF family that is susceptible to splicing modulation in a tumorigenic context, generating distinct isoforms. We next sought to explore the clinical and functional relevance of *ZNF217-ΔE4* expression in breast cancer.

Using a primer pair targeting exon 3, we have previously demonstrated that the *ZNF217* mRNA expression level of a breast tumor is informative and provides a powerful biomarker of poor prognosis, in particular in the Luminal subclass (5, 7). The present pilot retrospective study with 10-years follow-up provided evidence that the novel *ZNF217-WT-ΔE4* gene expression signature, which combines the expression of the two isoforms, retained clinical significance, and was significantly associated with shorter RFS in the Luminal subclass. The *ZNF217-WT-ΔE4* signature is thus capable of resuming the prognostic value of *ZNF217-E3* mRNA levels by allowing the stratification of the “good prognosis” Luminal subclass into good or poor/intermediate outcome. When considering the “good prognosis” SBRI+II subgroup, again the ZNF217-WT-ΔE4 signature could be used to refine the categorization of patients into good or poor/intermediate outcome. Our gene expression signature, however, was not informative in the “bad prognosis” subclasses such as HER2-enriched, Triple-negative, or SBRIII subgroups. Importantly, our univariate and multivariate analyses conducted in the Luminal subclass demonstrated that the prognostic value of the *ZNF217-WT-ΔE4* dual signature was more powerful than the expression levels of each isoform only, or than any of the usual clinical prognostic parameters. Altogether, our pilot retrospective study indicates the need to assess the mRNA expression levels of the two ZNF217 isoforms to obtain the most powerful prognostic biomarker value. Regarding the TCGA RNAseq data, the high proportion of censored cases (90%) prevented us from performing univariate analysis. While future work on an independent cohort is needed to confirm our findings, the results of our clinical investigation strongly suggest that the protein encoded by ZNF217-ΔE4 has a functional role.

Our study additionally pinpoints the existence of intricate molecular mechanisms involving ZNF217-WT and ZNF217-ΔE4. First, both the RNA-seq and RT-qPCR investigations, conducted on independent primary breast tumor cohorts, identified a positive correlation between *ZNF217-ΔE4* and *ZNF217-WT* mRNA levels. Second, our *in vitro* data revealed that ectopic overexpression of the ZNF217-ΔE4 isoform in breast cancer cells leads to: (i) increased endogenous mRNA and protein levels of the ZNF217-WT isoform; and (ii) features of cell aggressiveness (increased cell proliferation, increased anchorage-independent growth and resistance to paclitaxel) previously described when ZNF217-WT is ectopically overexpressed (4, 5). Our study thus indicates that the ZNF217-ΔE4 isoform triggers, directly or indirectly, a poor phenotype in breast cancer cells that may involve dysregulated ZNF217-WT expression levels. Little is known regarding the molecular mechanisms governing *ZNF217* transcription. Converging epigenetic data, however, show that the methylation status at CpG sites within the *ZNF217* locus correlates with inverse *ZNF217* expression levels (7, 15–18). Importantly, our methylome investigation revealed that constitutive expression of the ZNF217-ΔE4 isoform (but not of ZNF217-WT) is paired with decreased DNA methylation status on three CpG sites located on a key exon on the *ZNF217* gene. In a case-control study involving 1,083 blood samples (healthy women *versus* breast cancer patients), lack of methylation of the same *ZNF217* exon predicted increased breast cancer risk (26), allowing the authors to propose the DNA methylation status at this *ZNF217* locus to be a surrogate biomarker of breast cancer risk. We thus propose a model where the ZNF217-ΔE4 isoform plays a biologically relevant role in breast cancer, at least by impacting epigenetic-driven mechanisms governing the expression of ZNF217-WT. In future work, it would thus be of great interest to investigate the expression levels of both *ZNF217-ΔE4* and *ZNF217-WT* as well as the DNA methylation status of the *ZNF217* gene in breast cancer tissues from patients.

The predicted amino acid (aa) sequence encoded by the ZNF217-ΔE4 transcript shows that it shares a common region (aa 1 to 1012) with the ZNF217-WT protein, but the C-terminal region (49 aa) encoded by exon 5 is different from that present in ZNF217-WT protein and encoded by most of exon 4 (36 aa). The C-terminal region of ZNF217 is of the greatest interest because: (i) it possesses the transcriptional repressor domain (27) and a proline-rich domain (2) that might be involved in the ZNF217’s transcriptional activator properties; and (ii) it binds to CtBP1and CtBP2 transcriptional co-repressors (27, 28) and to the ER, modulating ER-signaling (7). Future work is thus required to determine how the ZNF217-ΔE4 interferes with the epigenetic machinery and whether it retains any transcriptional activity.

Little is known about the molecular mechanisms governing ASE in specific biological contexts. However, while the detailed mechanisms are still elusive, many recent studies have revealed that RNA N6-methyladenosine (m^6^A) modification serves as a mark for the recruitment of splicing factors and then affects ASE (20, 29). Interestingly, one elegant study has deciphered that the ZFP217 transcription factor (the murine ortholog of the human ZNF217) catalyzes m^6^A methylation at key transcripts, of which was the ZFP217 RNA itself (at exon3 and exon 5) (30). One thus cannot exclude that such ZNF217-WT-driven mechanisms may control splice events leading to the production of the ZNF217-ΔE4 isoform.

Overall, our new findings have important medical applications. Indeed, we have provided insights into the biological and clinical significance of the novel exon4-skipping ZNF217 isoform in breast cancer. Our pilot retrospective analysis emphasized the need to assess both *ZNF217-WT* and *ZNF217-ΔE4* expression levels to obtain the most powerful biomarker value, and suggested a key biological function of the encoded ZNF217-ΔE4 protein isoform. Our *in vitro* data supported our clinical data and reported for the first time that the ZNF217-ΔE4 protein drives cell aggressiveness and increased ZNF217-WT expression levels. In conclusion, our data highlight that assessing the expression levels of *ZNF217-WT* and *ZNF217-ΔE4* isoforms may serve as a novel prognostic biomarker allowing a better stratification of breast cancers with good prognosis and helping clinicians in therapeutic decisions. Future studies on the biological activities of ZNF217-ΔE4 protein and its interplay with ZNF217-WT will lead to a better understanding of ZNF217 oncogenic machinery.

## Data Availability Statement

Publicly available datasets were analyzed in this study. This data can be found here: https://www.nature.com/articles/nature11412.

## Ethics Statement

This study has been approved by the local ethics committee (CRB Centre Léon Bérard, France). The CRB Centre Léon Bérard is quality certified according to NFS96-900 French standard and ISO 9001 for clinical trials, ensuring scientific rigor for sample conservation, traceability, and quality, as well as ethical rules observance and defined rules for transferring samples for research purposes (Ministry of Health for activities authorization n◦ AC-2019-3426 and DC-2008-99). The material used in the study has been collected in agreement with all applicable laws, rules, and requests of French and European government authorities. All subjects gave written informed consent. The patients/participants provided their written informed consent to participate in this study.

## Author Contributions

BG and LP performed the biostatistical analyses of the RNAseq data. AB validated the *ZNF217* isoforms specific primers and performed *in vitro* experiments. DL and PAC performed the biostatistical analyses of the 107-breast tumor cohort. AW and JL performed and analyzed the methylome study. JV and JS performed the pyrosequencing experiments and analysis. JV and PC participated in scientific discussions. DL, BG, and PAC wrote the manuscript. PAC designed and supervised the study. All authors contributed to the article and approved the submitted version.

## Funding

This research program was supported by grants from the Pack Ambition International 2020 (Région Rhône-Alpes, France) and the French Ligue Contre le Cancer (committee 71). DL was funded by France Excellence Scholarship Program of the French Embassy in Vietnam. AB was supported by a Ph.D. grant from the French Ligue Nationale contre le Cancer. BG and LP were financed by the 2018-2.1.17-TET-KR-00001 and 2018-1.3.1-VKE-2018-00032 grants and the Higher Education Institutional Excellence Programme (2020-4.1.1.-TKP2020) of the Ministry for Innovation and Technology in Hungary, within the framework of the Bionic thematic programme of the Semmelweis University.

## Conflict of Interest

The authors declare that the research was conducted in the absence of any commercial or financial relationships that could be construed as a potential conflict of interest.

## Supplementary Material

Supplementary Figure 1 The sequence-specific primers for *ZNF217-WT (E3-E4)* and *ZNF217-ΔE4 (E3-E5)* isoforms with high specificity and sensitivity.**(A)** Binding sites of the primer pair 1, 2, and 3 used to specifically detect the *ZNF217-E3, ZNF217-WT (E3-E4)*, and *ZNF217-ΔE4 (E3-E5)* isoforms, respectively. The primer pair 1 hybridized within the sequence of *ZNF217*’s exon 3. The forward primer of pair 2 hybridized onto the exon 3 - exon 4 junction of *ZNF217-WT (E3-E4)* isoform. The reverse primer of pair 3 hybridized onto the exon 3 – exon 5 junction of ZNF217-ΔE4 (E3-E5) isoform. **(B)** RT-qPCR detection of *ZNF217* isoforms in mixtures containing 1,000 copies of different ratios of pcDNA6-ZNF217-WT and/or pcDNA6-ZNF217-ΔE4 plasmids amplified by RT-qPCR using the primer pair 1 (black bar), primer pair 2 (grey bar), or primer pair 3 (white bar). All data represent as mean ± standard deviation of two independent experiments conducted in triplicate. Frequency of **(C)** *ZNF217-ΔE4 (E3-E5)* mRNA levels and of **(D)** *ZNF217-ΔE4 (E3-E5)* mRNA levels among the 107 primary breast tumors.

Supplementary Figure 2 The *ZNF217-WT-ΔE4* gene expression signature is of poor prognosis and is associated with shorter relapse-free survival in the Luminal breast cancer subclass. Kaplan-Meier analyses (univariate analyses) for RFS in Luminal subclass (ER+ and/or PR +) of **(A)** *ZNF217-*E3 mRNA expression levels; **(B)** *ZNF217-WT (E3-E4)* mRNA expression levels; **(C)** *ZNF217-ΔE4 (E3-E5)* mRNA expression levels; **(D)** the 3-groups *ZNF217-WT-ΔE4* gene expression signature defined by: (group 1), low *ZNF217-WT (E3-E4)* and low *ZNF217-ΔE4 (E3-E5)* mRNA levels; (group 2), high *ZNF217-WT (E3-E4)* or high *ZNF217-ΔE4 (E3-E5)* mRNA levels; (group 3), high *ZNF217-WT (E3-E4)* and high *ZNF217-ΔE4 (E3-E5)* mRNA levels; **(E)** *ZNF217-WT-ΔE4* gene expression signature. *p* < 0.05.

Supplementary Figure 3 Characterization of MDA-MB-231-ZNF217-WT cells. **(A)** RT-qPCR analysis of *ZNF217-WT (E3-E4)* mRNA expression levels in MDA-MB-231-ZNF217-WT cell line and MDA-MB-231-pcDNA6 control cells (mean ± SD from three independent experiments, *a.u.*, arbitrary units) ****p <* 0.001, in Student *t*-test. **(B)** Representative Western blot analysis of ZNF217 WT protein expression levels using Abcam #48133 (upper panel) and Covalab 2 antibodies (lower panel). **(C)** RT-qPCR analysis of *ZNF217-ΔE4 (E3-E5)* mRNA expression levels in MDA-MB-231-ZNF217-WT cell line and in MDA-MB-231-pcDNA6 control cells (mean ± SD from three independent experiments, *a.u.*, arbitrary units). **(D)** Representative Western blot analysis of ZNF217-ΔE4 expression levels in MDA-MB-231-ZNF217-WT cell line *versus* control cells using RM217 antibody.

## Abbreviations

ZNF217-WT, ZNF217 wild-type; ZNF217-ΔE4, exon 4-skipping ZNF217 isoform; E3, exon 3; E3-E4, exon 3 – exon 4 junction; E3 –E5, exon 3 – exon 5 junction; ER, Estrogen Receptor alpha; ER-, ER-negative; HER2, Human epidermal growth factor receptor 2; PR, Progesterone receptor; RT-qPCR, Real-Time quantitative Polymerase Chain Reaction; RFS, relapse free survival; ASE, alternative splicing events; KLF, Krüppel-like factor; AA, amino acids; SBR grade, Scarff-Bloom-Richardson grade.
