# Supplementary figures and images for: Exploring the Significance of the Exon 4-Skipping Isoform of the ZNF217 Oncogene in Breast Cancer
Source: Front Oncol. 2021 Jul 2;11:647269. doi: 10.3389/fonc.2021.647269 (PMC8283766; doi:10.3389/fonc.2021.647269)

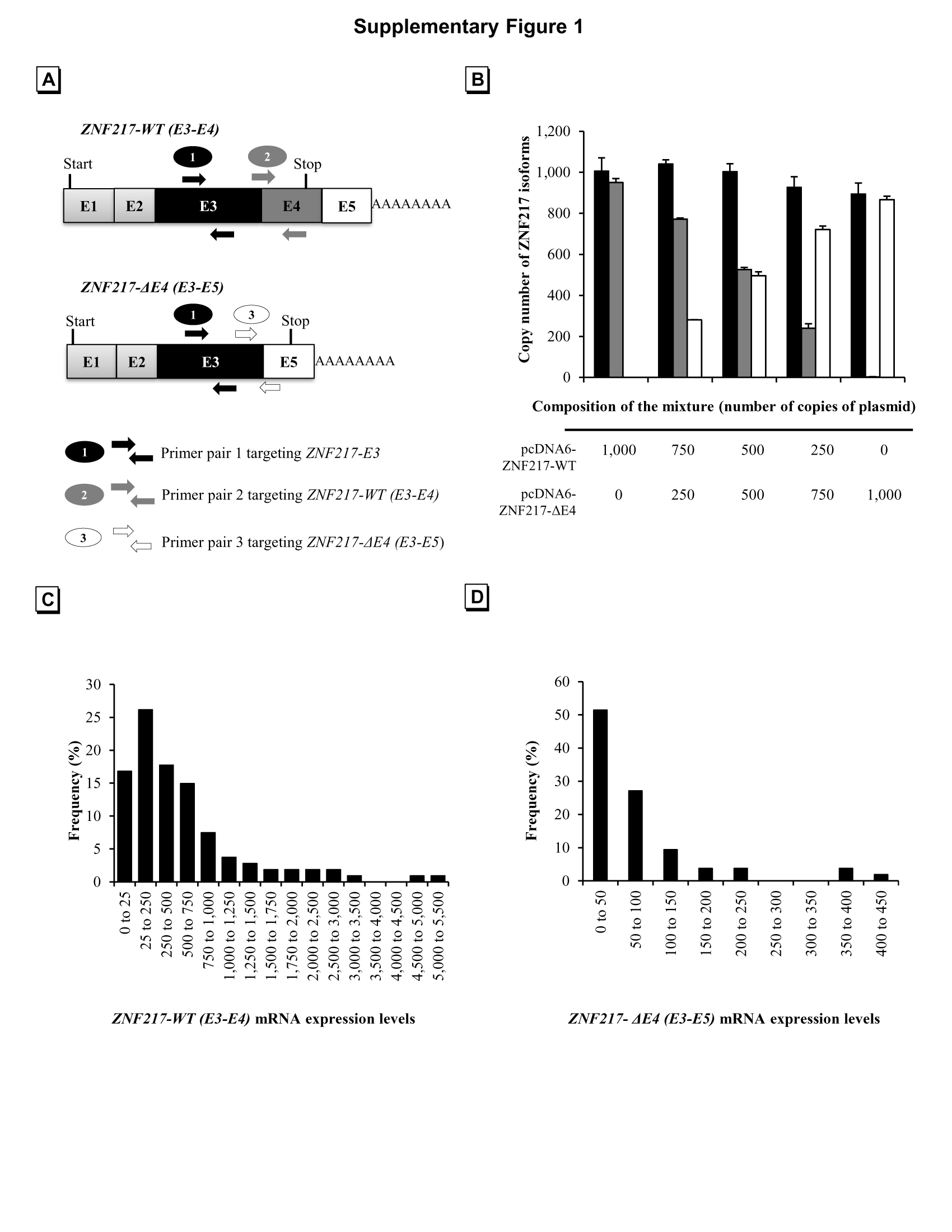

Supplement: Supplementary Figure 1 — The sequence-specific primers for ZNF217-WT (E3-E4) and ZNF217-ΔE4 (E3-E5) isoforms with high specificity and sensitivity.(A) Binding sites of the primer pair 1, 2, and 3 used to specifically detect the ZNF217-E3, ZNF217-WT (E3-E4), and ZNF217-ΔE4 (E3-E5) isoforms, respectively. The primer pair 1 hybridized within the sequence of ZNF217’s exon 3. The forward primer of pair 2 hybridized onto the exon 3 - exon 4 junction of ZNF217-WT (E3-E4) isoform. The reverse primer of pair 3 hybridized onto the exon 3 – exon 5 junction of ZNF217-ΔE4 (E3-E5) isoform. (B) RT-qPCR detection of ZNF217 isoforms in mixtures containing 1,000 copies of different ratios of pcDNA6-ZNF217-WT and/or pcDNA6-ZNF217-ΔE4 plasmids amplified by RT-qPCR using the primer pair 1 (black bar), primer pair 2 (grey bar), or primer pair 3 (white bar). All data represent as mean ± standard deviation of two independent experiments conducted in triplicate. Frequency of (C) ZNF217-ΔE4 (E3-E5) mRNA levels and of (D) ZNF217-ΔE4 (E3-E5) mRNA levels among the 107 primary breast tumors. [file Image_1.tif]

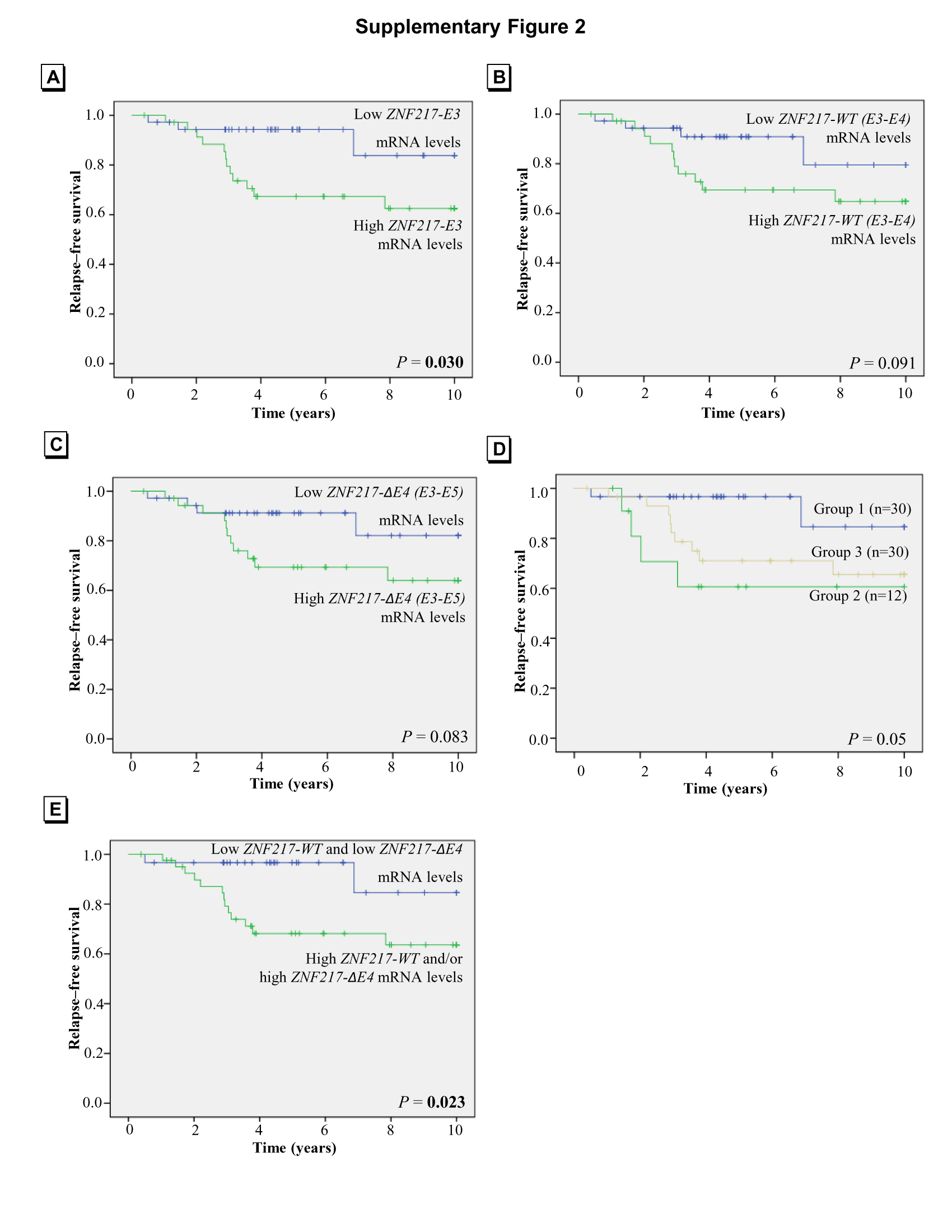

Supplement: Supplementary Figure 2 — The ZNF217-WT-ΔE4 gene expression signature is of poor prognosis and is associated with shorter relapse-free survival in the Luminal breast cancer subclass. Kaplan-Meier analyses (univariate analyses) for RFS in Luminal subclass (ER+ and/or PR +) of (A) ZNF217-E3 mRNA expression levels; (B) ZNF217-WT (E3-E4) mRNA expression levels; (C) ZNF217-ΔE4 (E3-E5) mRNA expression levels; (D) the 3-groups ZNF217-WT-ΔE4 gene expression signature defined by: (group 1), low ZNF217-WT (E3-E4) and low ZNF217-ΔE4 (E3-E5) mRNA levels; (group 2), high ZNF217-WT (E3-E4) or high ZNF217-ΔE4 (E3-E5) mRNA levels; (group 3), high ZNF217-WT (E3-E4) and high ZNF217-ΔE4 (E3-E5) mRNA levels; (E) ZNF217-WT-ΔE4 gene expression signature. p < 0.05. [file Image_2.tif]

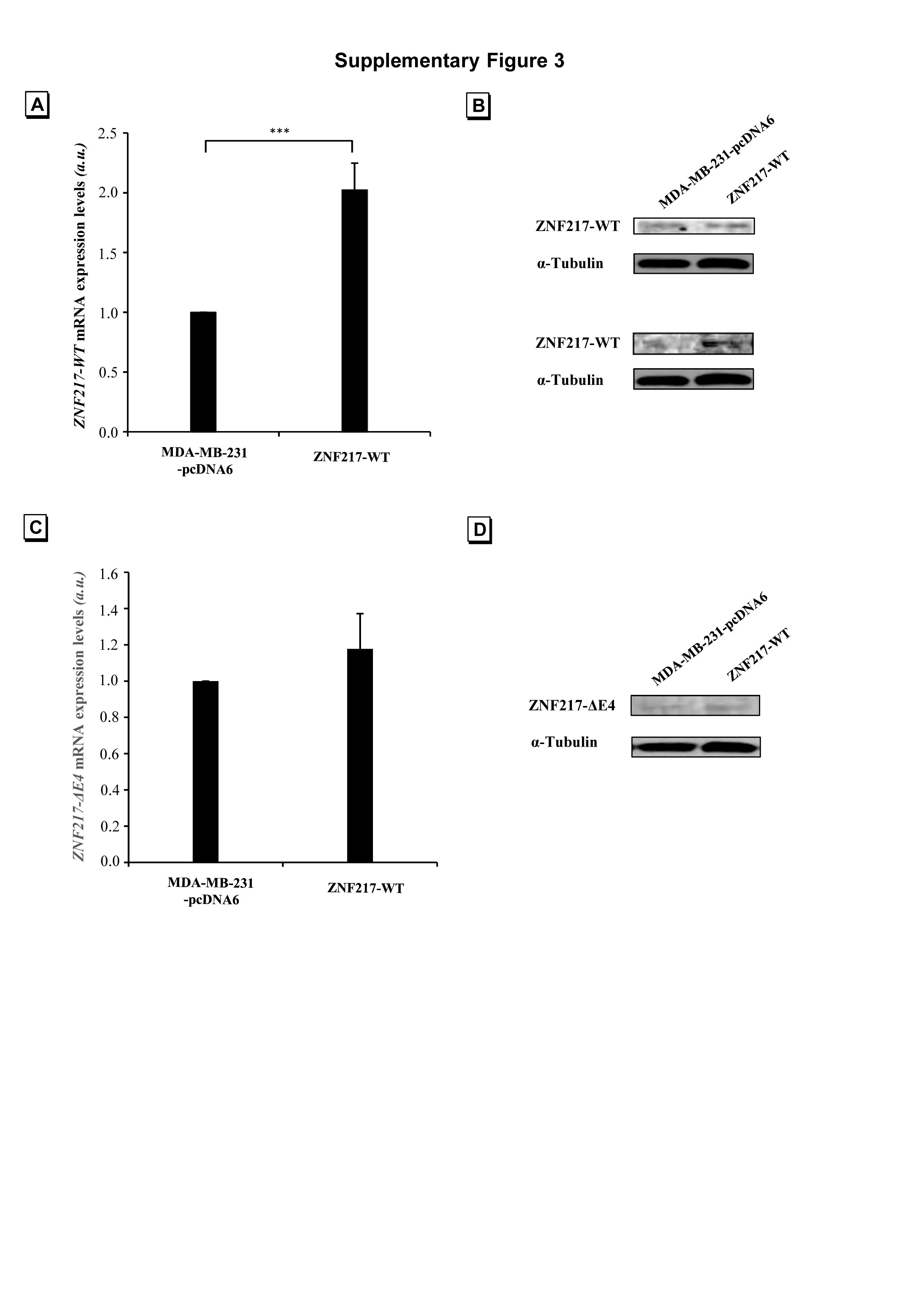

Supplement: Supplementary Figure 3 — Characterization of MDA-MB-231-ZNF217-WT cells. (A) RT-qPCR analysis of ZNF217-WT (E3-E4) mRNA expression levels in MDA-MB-231-ZNF217-WT cell line and MDA-MB-231-pcDNA6 control cells (mean ± SD from three independent experiments, a.u., arbitrary units) ***p < 0.001, in Student t-test. (B) Representative Western blot analysis of ZNF217 WT protein expression levels using Abcam #48133 (upper panel) and Covalab 2 antibodies (lower panel). (C) RT-qPCR analysis of ZNF217-ΔE4 (E3-E5) mRNA expression levels in MDA-MB-231-ZNF217-WT cell line and in MDA-MB-231-pcDNA6 control cells (mean ± SD from three independent experiments, a.u., arbitrary units). (D) Representative Western blot analysis of ZNF217-ΔE4 expression levels in MDA-MB-231-ZNF217-WT cell line versus control cells using RM217 antibody. [file Image_3.tif]
