# Supplementary material for: Exploring the Significance of the Exon 4-Skipping Isoform of the ZNF217 Oncogene in Breast Cancer
Source: Front Oncol. 2021 Jul 2;11:647269. doi: 10.3389/fonc.2021.647269 (PMC8283766; doi:10.3389/fonc.2021.647269)
Supplement: Supplementary file 4 [file DataSheet_1.pdf]

Supplementary Table S1. Characteristics of the 107 patients with primary breast cancers

	Number of patients	%
Age Diagnostic (years)		
≤ 50	53	49.5
> 50	54	50.5
SBR grade[a,b]		
I + II	45	42.5
III	61	57.5
Lymph node status		
Node negative	17	15.9
Node positive	90	84.1
Macroscopic tumor size[b]		
≤ 30 mm	39	36.8
> 30 mm	67	63.2
Estrogen Receptor status[c]		
Positive	64	59.8
Negative	43	40.2
Progesterone Receptor status[c]		
Positive	66	61.7
Negative	41	38.3
HER2 status[b,c]		
Positive	28	26.4
Negative	78	73.6

[a] Scarff-Bloom-Richardson classification.

[b] Information available for 106 patients.

[c] Measured by immunohistochemistry.

Supplementary Table S2. Univariate analyses of clinical parameters with regards to relapse-free survival

	**All tumor samples** (n = 107)			**Luminal subclass** [d] (n = 72)		
	HR[a]	95 % CI[b]	*p*[c]	HR[a]	95 % CI[b]	*p*[c]
Age (≤ 50 y old; >50 y old)	1.09	0.27-1.48	NS (0.290)	0.56	0.24-1.89	NS (0.450)
Histologic grade [e] (SBRI + II; SBRIII)	2.00	0.24-1.25	NS (0.150)	1.77	0.15-1.47	NS (0.183)
Lymph node status (negative; positive)	0.59	0.41-7.53	NS (0.440)	0.003	0.24-4.66	NS (0.953)
Macroscopic tumor size [e] (≤30 mm; >30 mm)	2.15	0.76-5.60	NS (0.142)	2.38	0.68-13.61	NS (0.123)
Estrogen-receptor status (positive; negative)	1.71	0.75-3.89	NS (0.190)	5.00	1.09-10.76	0.025
Progesterone-receptor status (positive; negative)	1.26	0.70-3.62	NS (0.260)	4.57	1.02-13.56	0.032
HER2 status [e] (negative; positive)	2.05	0.78-4.32	NS (0.150)	0.098	0.34-4.39	NS (0.754)

[a] HR, Hazard ratio.

[b] 95% CI, 95% confidence interval.

[c] *p* was considered significant when $p < 0.05$. NS, not significant.

[d] Luminal subclass (ER+ and/or PR+) was determined using immunohistochemistry (ER, PR) according to the St Gallen recommendation (12-14).

[e] Information available for 106 patients.

Supplementary Table S3. Statistical comparison of *ZNF217-E3*, *ZNF217-WT (E3-E4)*, *ZNF217-ΔE4 (E3-E5)* and *ZNF217-WT-ΔE4* expression levels among classical prognotic parameters (Age, Lymph node status, Macroscopic tumor size, SBR grade, Estrogen receptor status, Progesterone receptor status, HER2 status) in the whole cohort (n=107)

	ZNF217-E3 mRNA expression levels			*ZNF217-WT (E3-E4)* mRNA expression levels			*ZNF217-ΔE4 (E3-E5)* mRNA expression levels			*ZNF217-WT-ΔE4* signature		
	Low	High	*p**	Low	High	*p**	Low	High	*p**	Low	High	*p**
Age												
≤ 50 years (n = 53)	25	28	NS	26	27	NS	25	28	NS	20	33	NS
> 50 years (n = 54)	28	26	0.628	28	26	0.772	29	25	0.499	24	30	0.481
Lymph node												
positive (n = 90)	42	48	NS	42	48	NS	42	48	NS	35	55	NS
negative (n = 17)	11	6	0.172	12	5	0.070	12	5	0.070	9	8	0.280
Macroscopic tumor size[a]												
> 30 mm (n = 67)	30	37	NS	30	37	NS	32	35	NS	25	42	NS
≤ 30 mm (n = 39)	23	16	0.159	24	15	0.096	22	17	0.390	19	20	0.250
SBR Grade [a]												
I + II (n = 45)	18	27	NS	15	30	0.002	19	26	NS	13	32	0.024
III (n = 61)	35	26	0.077	39	22		35	26	0.123	31	30	

* P-value (χ2 test) was considered significant when P < 0.05.

[a] Information available for 106 patients.

Supplementary Table S3 (continued).

	ZNF217-E3 mRNA expression levels			*ZNF217-WT (E3-E4)* mRNA expression levels			*ZNF217-ΔE4 (E3-E5)* mRNA expression levels			*ZNF217-WT-ΔE4* signature		
	Low	High	*p**	Low	High	*p**	Low	High	*p**	Low	High	*p**
Estrogen receptor positive (n = 64)	32	32	NS 0.906	28	36	NS 0.090	34	30	NS 0.502	26	38	NS 0.899
negative (n = 43)	21	22		26	17		20	23		18	25	
Progesterone receptor positive (n = 66)	32	34	NS 0.783	29	37	NS 0.087	34	32	NS 0.783	26	40	NS 0.645
negative (n = 41)	21	20		25	16		20	21		18	23	
HER2[a] positive (n = 28)	14	14	NS 1.000	15	13	NS 0.746	12	16	NS 0.318	12	16	NS 0.866
negative (n = 78)	39	39		39	39		42	36		32	46	

* P-value ($\chi 2$ test) was considered significant when $P < 0.05$.

[a] Information available for 106 patients.
